# Digitalization of hydraulic rotary drilling process for continuously mechanical profiling of siliciclastic sedimentary rocks

**DOI:** 10.1038/s41598-023-30837-z

**Published:** 2023-03-06

**Authors:** X. F. Wang, Z. J. Zhang, W. V. Yue, Z. Q. Yue

**Affiliations:** 1 Department of Civil Engineering, China University of Geosciences (Beijing), Beijing, People’s Republic of China; 2 Department of Geotechnical Engineering, Tongji University, Shanghai, People’s Republic of China; 3 Department of Civil Engineering, The University of Hong Kong, Hong Kong, People’s Republic of China

**Keywords:** Stratigraphy, Sedimentology

## Abstract

Hydraulic rotary drilling can offer the essential information and core samplesa for the researches on solid earth. Recording the factual field drilling data and analyzing the hydraulic rotary coring process are challenging yet promising to utilize the massive drilling information in geophysics and geology. This paper adopts the drilling process monitoring (DPM) technique and records the four parameters of displacement, thrust pressure, upward pressure, and rotation speed in real-time series for profiling the siliciclastic sedimentary rocks along 108 m deep drillhole. The digitalization results with 107 linear zones represent the spatial distribution of drilled geomaterials including superficial deposits (fill, loess, gravelly soil), mudstone, silty mudstone, gritstone, and fine sandstone. The constant drilling speeds varying from 0.018 to 1.905 m/min present the in-situ coring resistance of drilled geomaterials. Furthermore, the constant drilling speeds can identify the strength quality of soils to hard rocks. The thickness distributions of the six basic strength quality grades are presented for all the sedimentary rocks and each individual type of the seven soil and rocks. The in-situ strength profile determined in this paper can be used to assess and evaluate the in-situ mechanical behavior of geomaterial along the drillhole and can provide a new mechanical-based assessment for determining the spatial distribution of geological strata and structures in subsurface. They are important since the same stratum at different depths can have different mechanical behavior. The results provide a novel quantitative measurement for continuously in-situ mechanical profiling by digital drilling data. The findings of the paper can offer a new and effective method for refinement and upgrading of in-situ ground investigation, and can provide researchers and engineers with a novel tool and valuable reference to digitize and utilize factual data of current drilling projects.

## Introduction

Drilling, particularly hydraulic rotary coring drilling, is a common, essential, and important operation to offer the core samples and associated information for the geophysical and geological researches on solid earth^1^. The associated drilling information has become the focus of increasingly cross-disciplinary studies within the growing digitalization of solid earth observatories all over the world^2^. Flinchum et al. used the seismic refraction data and physical state of core samples from hydraulic rotary coring process to infer the subsurface structure under a granite ridge^3^. Allen et al. revealed the presence of a 30 m thick alteration zone overprinting both the fault core and damage zone by laboratory test results of core samples along one 100.6 m drillhole^4^. The laboratory measurements on core samples extracted from drillholes are widely used to quantify the downhole variations on the mechanical and geophysical properties^5–7^.

However, the laboratory core measurements and some in-situ tests for many drillholes can be prohibitively expensive and logistically challenging, particularly under some complex geological environments where geophysical studies are often focused^3^. In fact, drilling itself can also be considered as an in-situ measurement for geomaterial properties^8–10^. Massive factual data of drilling parameters such as drilling speed (or penetration rate), as by-products of drilling process, have not been collected and utilized. It may contains the mechanical and geophysical information. Digitalization of drilling process with factual data is challenging yet promising to research in the solid earth.

Several researchers focused on the studies of drilling information. Rizzo et al. used the digital electrical signals from the drillhole to identify the hydraulic conductivity distribution^11^. In-situ strain information was monitored and analyzed by the four-gauge borehole strainmeters (FGBS)^12^. The measurement-while-drilling (MWD) technology was used to measure the drilling parameters of penetration rate, thrust pressure, and rotational speed in petroleum and mining science and engineering^13–15^. Yue et al. invented the drilling process monitoring (DPM) technique and developed the real-time series method in factual data measurement and analyses^10,16^.

Recently, more and more researchers pay attention to the drilling data in real-time series. The real-time drilling data can identify the weak zones in volcanic rock^17,18^ and profile the soil to rock strength in loess tableland^19^. Wang et al. studied the method for measuring rock mass characteristics by laboratorial real-time drilling data^20^. He et al. proposed an empirical method to determine the rock mass mechanical properties and the rockburst proneness prediction^21,22^. Arnø et al. proposed the estimation of rock density from real-time drilling data using deep learning method^23^. Most of the previous researches studied the drilling information by laboratory test data and empirical methods. The quantitative study for continuously and accurately in-situ mechanical profiling by factual filed data is still very limited. Further studies on factual field data of current drilling projects and detailed analysis of the hydraulic rotary coring process for the refinement and upgrading of in-situ ground investigation are motivated and carried out in this paper.

In this paper, the digitalization of the hydraulic rotary drilling process is presented to fill the gap in geophysical and geological drilling projects. Digital factual data in real-time series is recorded from the hydraulic rotary coring process along the 108 m drillhole. The results show that the factual drilling data can continuously present a quantitative description of the in-situ mechanical profiling of geomaterial resistance to drilling force and the estimated uniaxial compressive strength from superficial deposits to siliciclastic sedimentary rocks along the drillhole. The geomaterial resistance to drilling force was determined using the drilling speed that was accurately obtained in this paper from the digital DPM technique. Such continuous in-situ strength profiles are important when assessing the stability and possible collapse of the surrounding soils and rocks around the drillhole. They can provide additional factual and in-situ data to the coring results along the drillhole. Particularly, these information become crucial when there are missing cores along the drillhole depth. The paper demonstrates the framework of a new in-situ test methodology based on drilling data aimed to upgrade the current ground investigation methods and rock quality classifications.

## Hydraulic rotary drilling equipped with digitalization instrument at site

The project of *Comprehensive Geological Survey in Yan’an city* by China Geological Survey aims to profile the mechanical properties with digital factual data for developing urban underground space^24^. The city of Yan’an is located in mountainous loess plateau region. It is a typical problematic area that the conversion of rock to soil is driven by diverse physical, chemical, and biological processes within a spatially variable and complex zone spanning the top 10–100 m in this region^25^. Fast, effective, and continuous geotechnical investigation is essential for the assessment and utilization of the underground spaces^26^. Therefore, the drilling process monitoring (DPM) technology is used to continuously provide the valuable reference of in-situ mechanical profile by digital factual data in the project.

**Figure 1:**
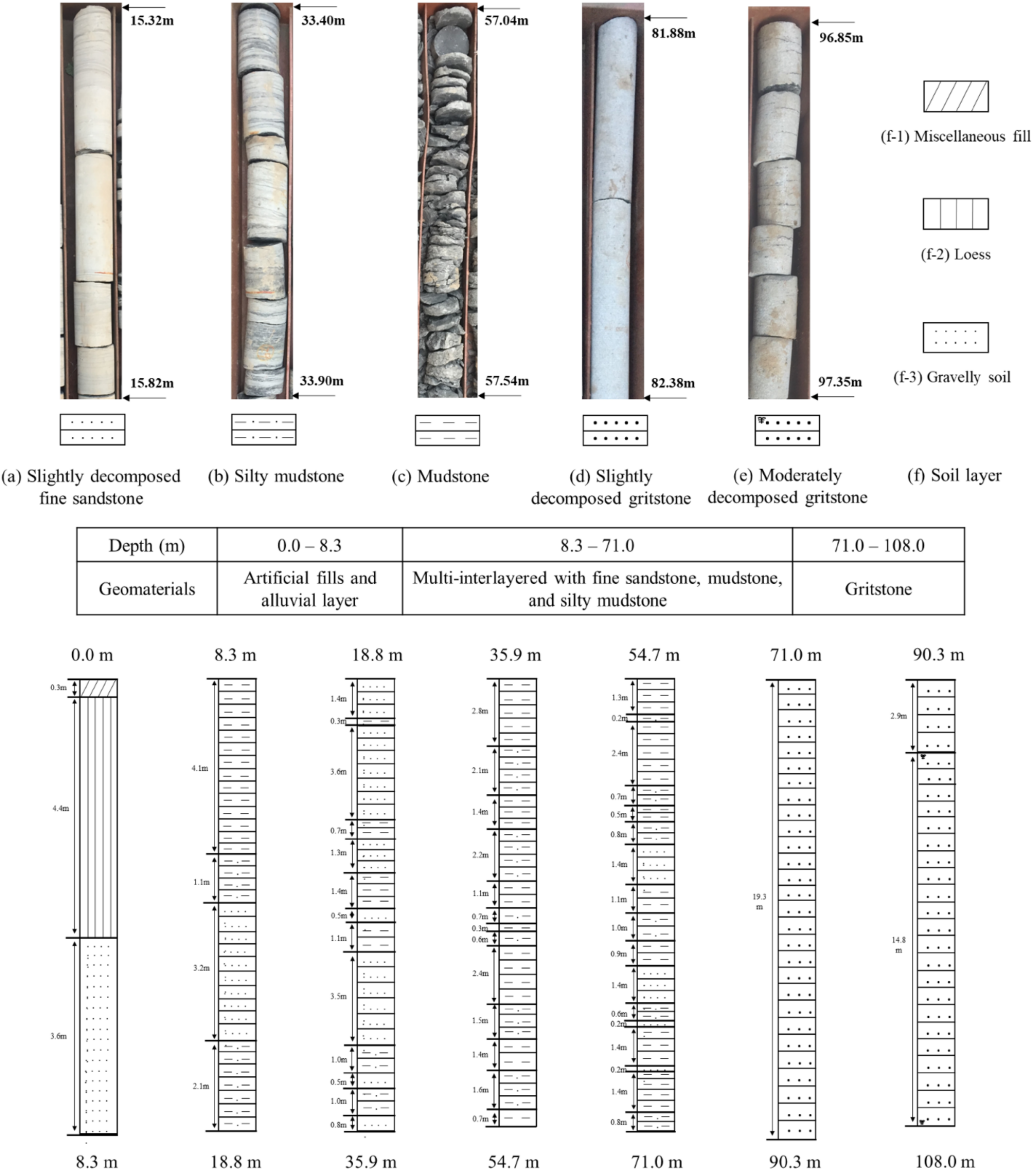
Eight types of strata along this 108 m drillhole with selected rock core samples and corresponding standard stratigraphic column by manual site logging.

The coring hydraulic rotary drilling project is located at the Baota district in the city of Yan’an with the GPS coordinate (36° 37′ 48′′ N, 109° 22′ 48′′ E). The drillhole depth is 108.0 m by site loggings, and the geomaterials along the drillhole and corresponding standard stratigraphic column are shown in Fig. 1. In order to improve efficiency at the site, the core samples were only collected from the rock layers. The accuracy of the stratum thickness by site loggings and core samples is 0.1 m and all the core recovery rates are above 90%. Based on the site records and manual loggings of core samples, the drillhole has a total of 51 strata for different ground soils and sedimentary rocks. The 51 strata correspond to the 3 layers of ground soils and the 48 rock layers, as shown in the standard stratigraphic column. The maximum value of the layer thickness is 22.2 m, it corresponds to the stratum of slightly decomposed gritstone. The minimum value is 0.2 m, it corresponds to the stratum of fine sandstone.

The manual site logging shows that the superficial deposits are composed by artificial fills (e.g., miscellaneous fill and loess) and alluvial layer (e.g., gravelly soil) from 0.0 to 8.3 m along the drillhole. The remaining part along the drillhole (8.3–108.0 m) belongs to the Yan’an formation with two members, consisting of siliciclastic sediments deposited in alluvial, lacustrine, and mire settings during the Middle Jurassic^24,25^. Core samples collected from Zaoyuan Member (8.3–71.0 m) are multi-interlayered with fine sandstone, mudstone, and silty mudstone. Samples collected from Baotashan Member (71.0–108.0 m) are dominated by gritstone.

**Figure 2:**
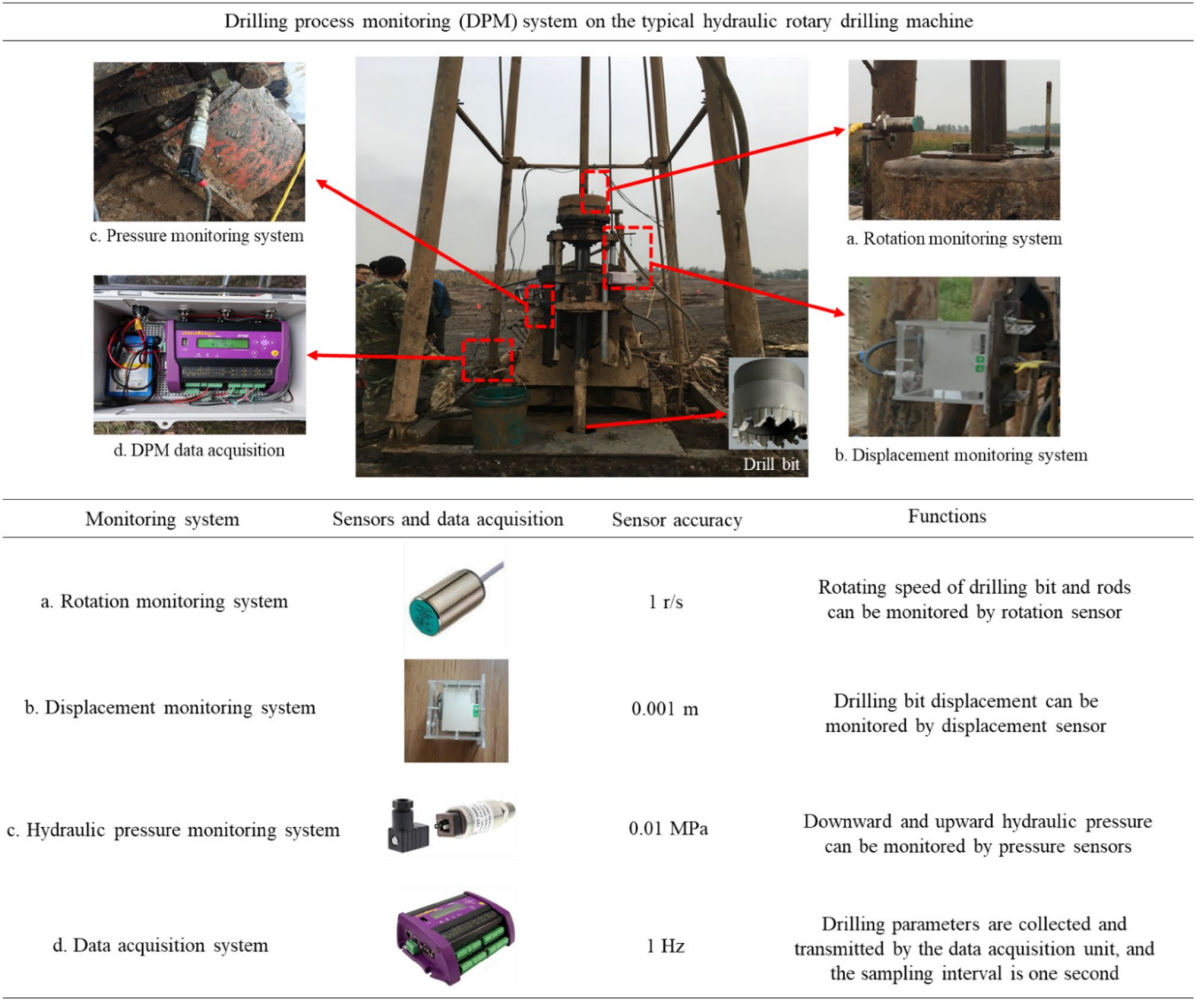
Schematic diagram of digitalization system.

The typical XY-1-type hydraulic rotary drilling machine and the polycrystalline diamond compact drill bit with 110-mm diameter are used for the drilling as shown in Fig. 2. The drilling method is wet drilling with fluid and the drilling fluid is bentonite slurry made of SM vegetable gum, bentonite, and water with the weight ratio 1:5:100.

To investigate the relationship between drilling parameters and mechanical profiling of drilled geomaterials, the data of displacement, thrust pressure, upward pressure, and rotating speed are monitored in this study. The digitalization instrument can be easily and non-destructively mounted onto the hydraulic rotary drilling machine. It can automatically, objectively, and continuously collect the digital factual data in real time series at site. The displacement is used for determining the drilling speed and the drillhole depth. The thrust pressure and rotating speed are used to understanding the drilling power. The upward pressure is used to determine the other sub-processes of the drilling work. During this drilling project, the digitalization system consists of the following main parts as shown in Fig. 2.

The rotation monitoring system (Fig. 2a) contains one electromagnetic revolution transducer, an induction rig to rotate with the drilling bit and rods, and the associated protection and fixture devices. The rotation of drilling bit in hydraulic rotary drilling provides horizontal force to grind the touched geomaterial surface. The rotation sensor accuracy is one revolution per second (60 revolutions per minute).

The displacement monitoring system (Fig. 2b) contains one swivel displacement transducer and the associated protection and fixture devices. The drill bit breaks the ground materials and advances into new ground materials below the bit. The system can record the drill bit displacement from the downward or upward displacement of the swivel drill chuck head along the two vertical hydraulic cylinders during the whole drilling process. The ram stroke length of the cylinders is the maximum vertical displacement distance for the drill chuck head, which is 500 mm in this case. The displacement sensor accuracy is 0.001 m.

The hydraulic pressure monitoring system (Fig. 2c) contains two pressure transducers. They are installed on the hydraulic pressure pipe for the thrust and upward force calculation. During the drilling process, the downward thrust provides the power to keep the bottom drill bit contacting geomaterial surface and cutting it while rotating and the upward force provides the power to move the drill chuck head upward along the two vertical hydraulic cylinders after finishing one ram stroke advancement. Furthermore, the drilling torque is a reaction effect of the thrust and rotation speed. This parameter is not a direct output parameter, and it cannot be shifted by the operators. So it is difficult and expensive to monitor drilling torque in the traditional drilling project for ground investigation at site. During drilling process, the torque can be regarded as a linear relation with thrust^27,28^.

The data acquisition system (Fig. 2d) controls the sampling of signals from the system above. It collects four signals simultaneously in the form of voltage output in real-time series and the time-sampling interval is 1 s in this case.

The monitoring and data acquisition systems are portable and convenient to be installed. The digitalization system can monitor and analyze the full drilling process without any side effects for either the drilling machine or the routine operations at site.

## Digitalization method for hydraulic rotary coring drillhole

### Individual drilling operations associated with full drilling process

The full hydraulic rotary drilling process consists of many roundtrips in time sequence due to the fact that the soil and rock core sample in the cylindrical tube sampler above the drill bit has to be retrieved once the tube sampler is full. Each roundtrip can be described as follows.

Step 1: the drill bit with the core sampler and several extension drill rods are inserted down to the bottom of the drillhole one by one.
Step 2: the bit-sampler and rods are rotated by the rig for warming up and stirring the mud slurry, which can facilitate the drilling effect.
Step 3: the drill bit starts to break the ground materials and advance into new ground materials below the bit, which will be stop once the sampler (coring) barrel is filled up with the soil or rock cores.
Step 4: all the drill rods and bit-sampler are retrieved back to the ground to collect the samples.

Some auxiliary operations (e.g., intermission or maintenance) may be needed during the above processes. These four sub-processes including (1) inserting the bit-sampler (barrel), (2) coring and filling the barrel with new ground materials, (3) retrieving the filled barrel back to the ground, and (4) collecting the core samples on the ground form a typical roundtrip. This roundtrip is repeated one by one for drilling deeper geomaterials until the target drilling depth is reached. Therefore, the number of roundtrips is mainly determined by the length of sampler (coring) barrel and the operations of drilling workers. In this case study, the length of sampler (coring) barrel was 4.15 m and a total of 26 roundtrips were used for completing the 108 m deep drillhole.

The original real-time factual drilling data (displacement, thrust pressure, upward pressure, and rotating speed) can be continuously collected by the digital system equipped on the hydraulic rotary drilling machine during the full drilling process, as shown in Fig. 2. According to the factual data and digitalization criterion, the typical coring hydraulic rotary drilling roundtrip can be divided into five individual drilling operations. They are the inserting process, the stirring process, the drilling process, the retrieving and extracting process, and the auxiliary process, respectively. Each operation is distinctive and correlated with the above control of operators.

### DPM drilling parameters associated with net drilling process

During the drilling process, the operators use the swivel chuck head to control the drill spindle which is connected to the drill rods and bit for coring the geomaterials. The chuck head can clutch the drill spindle to drill the new geomaterials. After finishing one ram stroke length mentioned above, the chuck head detaches the spindle to move back to the start level and then it can clutch the spindle again to move it downward. By this operation, the drill bit can drill deeper geomaterials with the new added drill rod to fill the coring barrel. Therefore, the drilling process has three parts: the coring part (or the net drilling part), the pulling drill chuck upward part, and the auxiliary operations.

The classical least-squares method is used to determine the drilling speed by factual data and the coefficient of determination *R*^2^ or* r*^2^ is usually used to measure the goodness of fit for the extent of the linearity. The minimum time interval of one linear zone is usually greater than 5 s.

**Figure 3:**
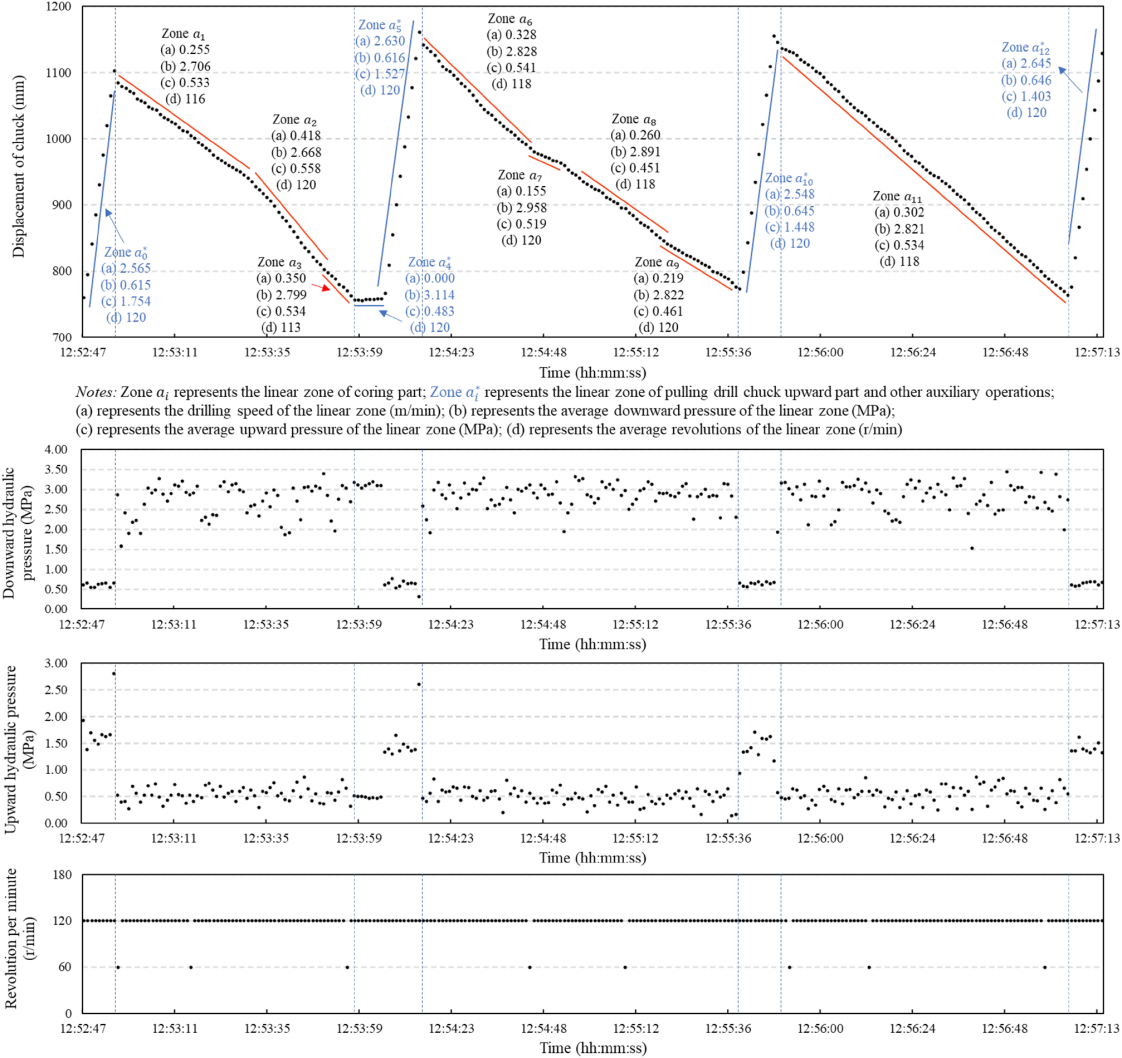
Real-time factual data for the drilling process of hydraulic rotary coring along this drillhole.

Figure 3 shows the details of one drilling process. It contains three coring parts and four pulling drill chuck upward parts. During the coring parts, the curve of the drill bit advancement depth versus the net drilling time can be expressed as a set of connected linear segments. Each linear zone has a constant slope gradient representing the constant drilling speed of a homogeneous geomaterial. In Fig. 3, eight linear zones (Zones ${a}_{1-3}$, Zones ${a}_{6-9}$, and Zones ${a}_{11}$) with different constant gradients are shown in the coring parts. The constant gradient of one linear zone is equal to the drilling speed of that linear zone. The drilling speeds of the eight linear zones in the coring part vary from 0.155 m/min (meter per minute) to 0.418 m/min. The corresponding average thrust pressure, average upward pressure, and average revolution of each zone in the coring part are set up to fluctuate in a small range of 2.688–2.958 MPa, 0.451–0.558 MPa, and 113–120 r/min (revolution per minute), respectively. The pulling drill chuck upward parts contain four zones (Zones ${a}_{0}^{*}$, ${a}_{5}^{*}$,${a}_{10}^{*}$, and ${a}_{12}^{*}$) with occasional auxiliary intermission (Zone ${a}_{4}^{*}$). The pulling speed increases to the range of 2.548–2.645 m/min, the average thrust pressure decreases to the range of 0.615–0.646 MPa, and the average upward pressure increases to the range of 1.403–1.754 MPa.

### Digitalization analysis results along the whole drillhole

**Figure 4:**
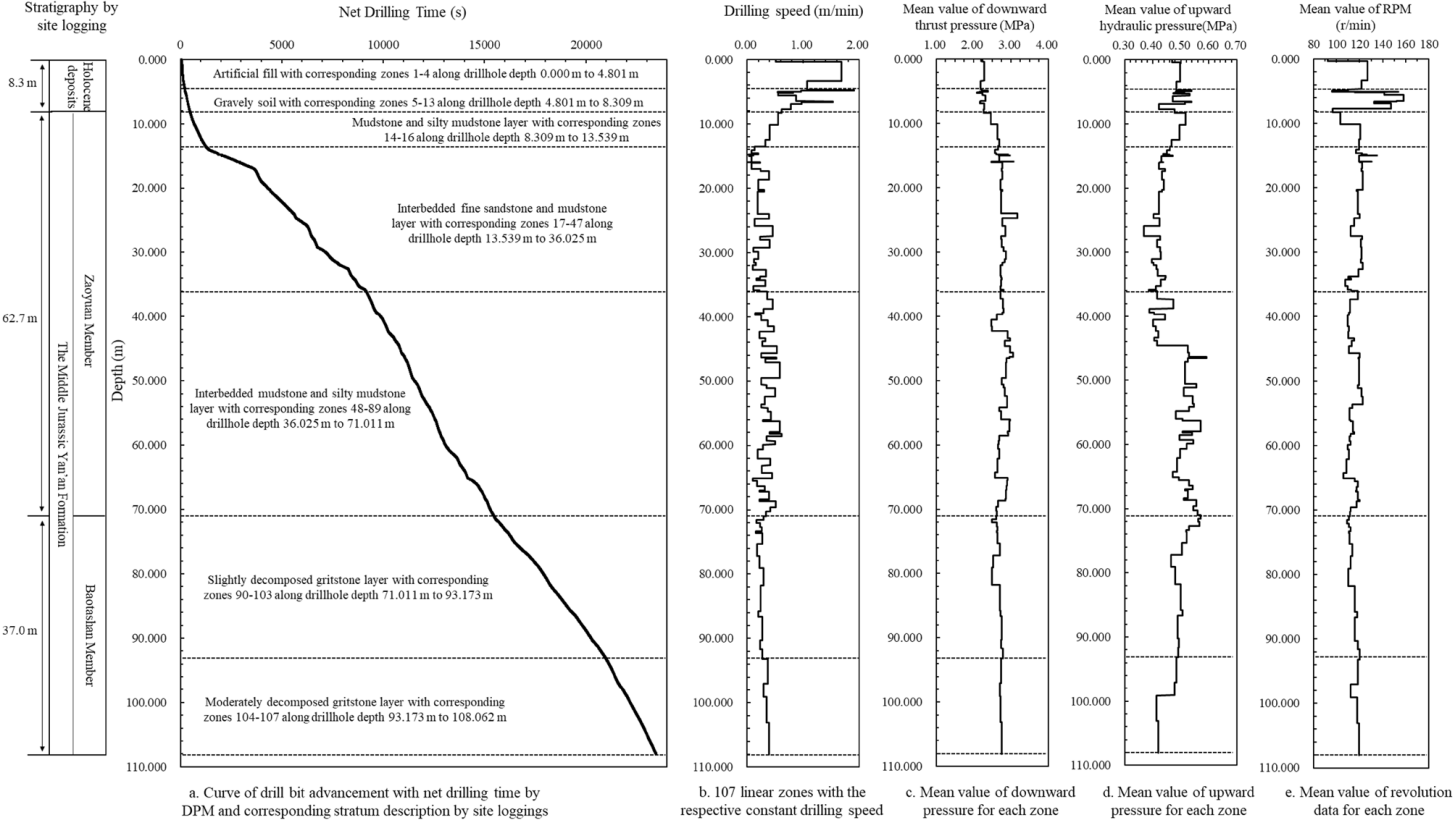
The curve of drill bit advancement with net drilling time and associated drilling parameters along the whole drilling depth by factual data.

Based on the screening method of individual drilling operations, the original factual data of the net drilling process can be obtained. Then the drilling parameters of the net drilling process along the whole drillhole depth is shown in Fig. 4. The drillhole depth measured by DPM is 108.062 m. Figure 4a,b show that the curve of drilling depth with net drilling time can be divided into 107 linear zones with respective constant drilling speeds. The drilling speed varies from 0.018 to 1.905 m/min. Each constant drilling speed zone represents one homogeneous geomaterial. The connection depth of two adjacent linear zones with different drilling speeds means the boundary of two different strata. The brief site loggings in Fig. 4a show all the strata of the drillhole with the corresponding drilling speed zones. The superficial deposits in Holocene correspond to the digitalization zones Nos. 1 to 13 along the drillhole depth 0.000–8.309 m, consisting of miscellaneous fill, loess, and gravelly soil. The Zaoyuan Member in the Middle Jurassic Yan’an Formation corresponds to the digitalization zones Nos. 14 to 89 along the drillhole depth 8.309–71.011 m, consisting of the multi-interlayered fine sandstone, mudstone, and silty mudstone. The Baotashan Member in the Middle Jurassic Yan’an Formation corresponds to the digitalization zones Nos. 90–103 along the drillhole depth 71.011–108.062 m, consisting of the gritstone. The detailed comparisons of digitalization results and traditional manual site loggings are discussed in the next section.

The associated drilling parameters of the net drilling process can be calculated for each drilling speed zone. Figure 4c shows the mean value of downward thrust pressure for each zone along the drillhole. As the routine operations for improving drilling efficiency, the thrust pressure in soil layer is fixed at the lower gear and the thrust pressure in rock layer is fixed at the higher gear. The mean values of thrust pressure for the soil strata (correspond to zones Nos. 1–13) fluctuate within a lower range from 2.096 to 2.382 MPa. The mean values of thrust pressure for the rock strata (correspond to zones Nos. 14–107) fluctuate within a higher range from 2.496 to 3.179 MPa. Figure 4d shows the mean value of upward pressure for each zone along the drillhole. As mentioned above, the upward pressure is fixed at the standby gear for preparing the subsequent action during the net drilling process. The mean values of upward fluctuate within the standby gear range from 0.368 to 0.591 MPa. Figure 4e shows the mean value of rotating speed for each zone along the drillhole. The main fluctuation range of revolution data at the routine working gear is from 105 to 120 r/min. Moreover, the operators elevated the rotation speed to a higher working gear for drilling efficiently at the gravelly soil layer as a routine method. So, the mean values of revolution data for drilling speed zone Nos. 5–13 are higher than others.

**Figure 5:**
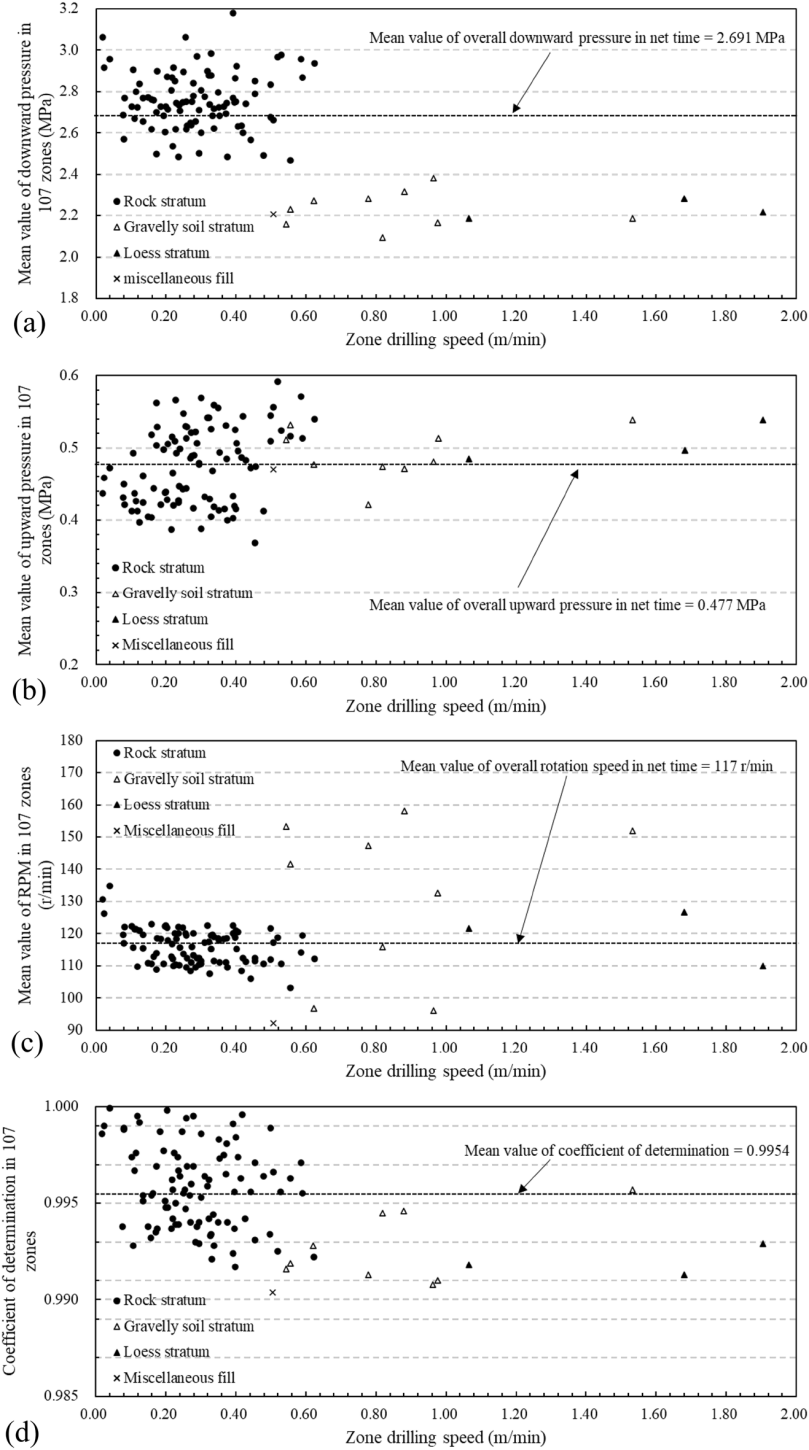
The relation between drilling speed of 107 depth zones and corresponding drilling parameters in the same zones (**a**) mean values of downward pressure, (**b**) mean values of upward pressure, (**c**) mean values of revolution, (**d**) coefficient of linear correlation.

In addition, the constant drilling speed with DPM method is determined without direct consideration of the possible effects for the thrust, rotation, and torque. Yue indicated that the normalized drilling speed with other drilling parameters is almost the same as the original drilling speed by DPM method^10^. In this drillhole, the effect of associated drilling parameters for constant drilling speed can be presented in Fig. 5.

In Fig. 5a, as the zone drilling speed of rock stratum increases, the mean values of downward thrust pressure are fixed to fluctuate within a certain range of high gear. As the zone drilling speed of soil stratum increases, the mean values of downward thrust pressure are fixed to fluctuate within the lower range. The similar relationships between zone drilling speed and associated drilling parameters are also shown in Fig. 5b,c. Figure 5d shows the coefficient of determination for the constant drilling speed of all 107 zones. The mean value of the coefficient of determination is 0.9954. According to Fig. 5, the associated drilling parameters fluctuate within a certain range with the increase of drilling speed, meanwhile, all the coefficient of determination for the increasing drilling speed are more than 0.99. The downward thrust pressure, upward pressure, and the rotation speed during the net drilling process have limited effects on the variations of the constant drilling speed for homogenous geomaterial zones. Therefore, the drilling speeds were mainly related to the drilled geomaterial properties since the applied thrust, rotation and torque had limited variations for different homogenous geomaterial zones in this case study.

According to the results in Fig. 4, each stratum can correspond to several constant mechanical zones (or constant drilling speed zones). It means that digitalization results can show the detailed coring resistant strength profiles and associated spatial distributions for one stratum by manual loggings. The similar drilling speeds of linear zones represent the geomaterials with similar in-site coring resistant strength profiles and the position of the curve gradient jump between any two connected linear zones can represent the interface boundaries of different in-site coring resistant strength profiles. The spatial distributions of constant mechanical zone with constant drilling speed (or drilling resistance) for profiling the different geomaterials by digitalization results are consistent with the distributions of core samples by site loggings. The strata thicknesses and drillhole depth by digitalization results have been verified by the corresponding manual loggings.

## Digitalization method for zoning the in-situ mechanical profiles

### In-situ mechanical zones with constant drilling speed for the different geomaterials along the drillhole

**Figure 6:**
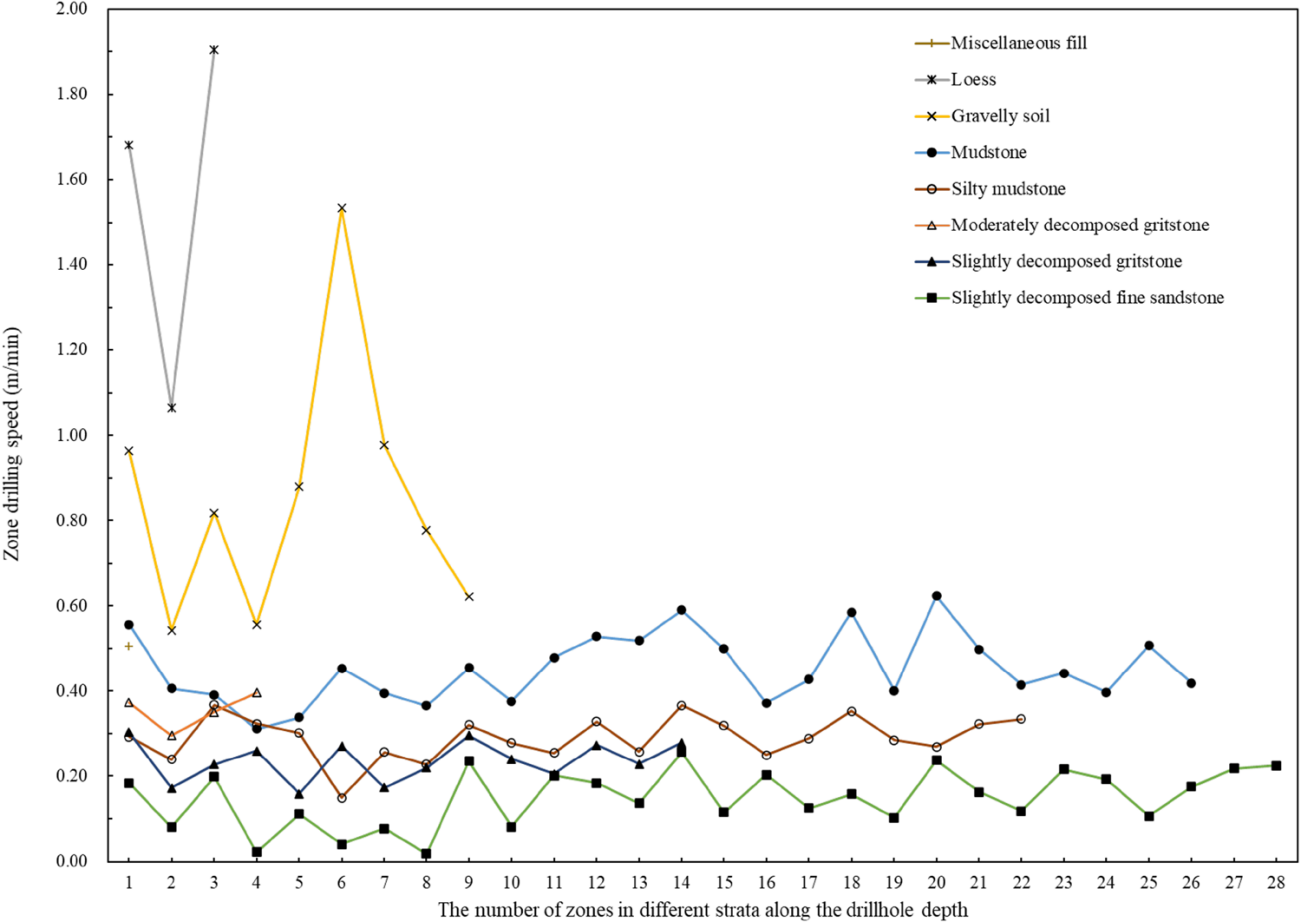
The number of constant mechanical zones in different strata with associated zone drilling speed.

According to the analysis results above, each constant mechanical zone with its constant drilling speed (or drilling resistance) corresponds to one homogeneous geomaterial. Consequently, the digitalization method can profile the coring-resistant strength of the soils to siliciclastic sedimentary rocks by factual data. A total of 107 constant mechanical zones can be obtained for the eight types of soils to siliciclastic sedimentary rocks. Figure 6 presents the number of zones in different strata with the associated drilling speed. For the soils to sedimentary rocks along the drillhole, the constant drilling speeds are from 0.018 to 1.905 m/min, the average and median values of 0.373 and 0.295 m/min, respectively. The soil layers have thirteen zones and the rock layers have ninety-four zones. The same geomaterial zones fluctuate within the similar range of drilling speed. Different geomaterials show different fluctuation ranges of drilling speed. Besides, the drilling speeds of soil layers fluctuate within a large range than those in rock layers. It indicates that the drilling speed of one rock layer is more stable than the drilling speed in one soil layer.

**Figure 7:**
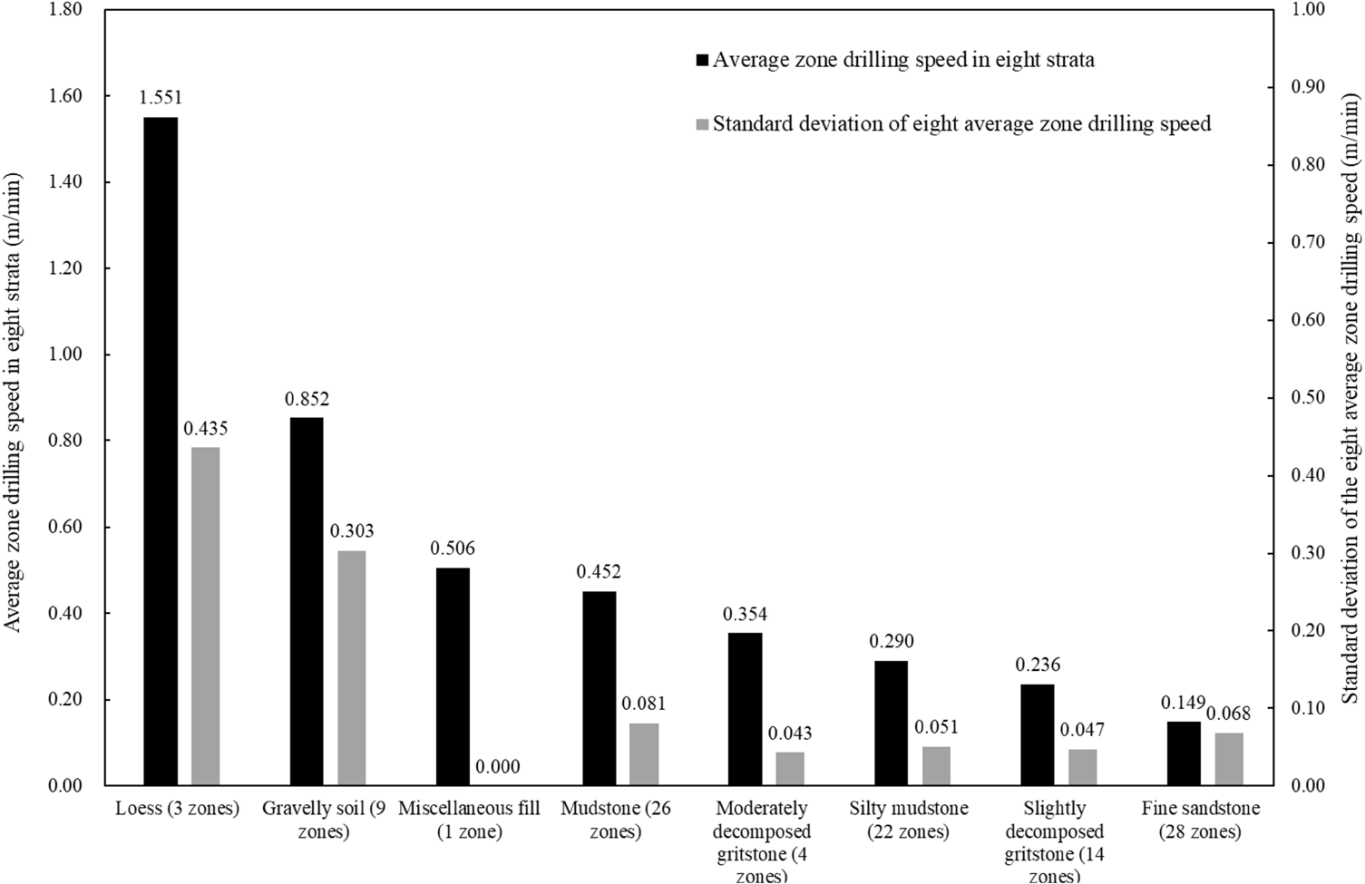
The average zone drilling speed and corresponding standard deviation of eight strata in the drillhole.

With the drilling speed of 107 zones in different stratum, the average zone drilling speeds of each geomaterial and the associated data can be calculated in Fig. 7 and Table 1. The average drilling speeds and corresponding standard deviations of loess and gravelly soil layers are higher than others. The average drilling speed of mudstone is the highest among the five kinds of rock layers. The average drilling speeds of the other four rock types are displayed in descending order and the lowest drilling speed corresponds to the fine sandstone. It is obvious that the zones with lower drilling speed represent a stronger or less easily drillable geomaterial.

**Table 1:** The zone drilling speed and zone thickness by digitalization results for eight strata.

Geomaterial	The average value of zone drilling speed (m/min)	The maximum value of zone drilling speed (m/min)	The minimum value of zone drilling speed (m/min)	The standard deviation (m/min)	The number of constant drilling speed zones
Loess	1.551	1.905	1.065	0.435	3
Gravelly soil	0.852	1.533	0.543	0.303	9
Miscellaneous fill	0.506	0.506	0.506	0.000	1
Mudstone	0.452	0.623	0.311	0.081	26
Moderately decomposed gritstone	0.354	0.396	0.295	0.043	4
Silty mudstone	0.290	0.367	0.149	0.051	22
Slightly decomposed gritstone	0.236	0.303	0.159	0.047	14
Slightly decomposed fine sandstone	0.149	0.257	0.018	0.068	28

**Figure 8:**
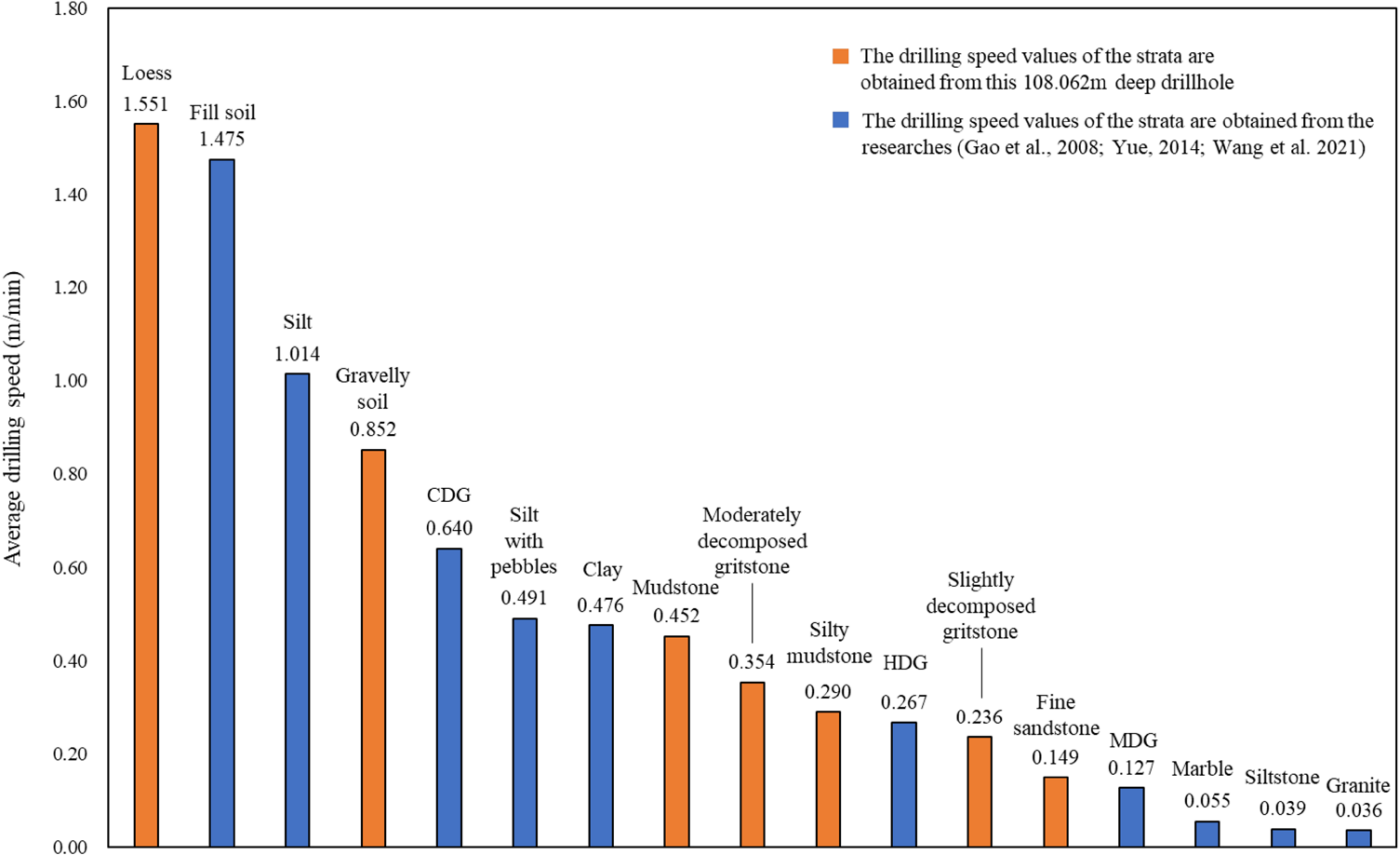
The average drilling speed for different geomaterials in the drillhole.

Furthermore, the average drilling speeds of different geomaterials along this drillhole are further compared with some other geomaterials with their corresponding average drilling speed in Fig. 8. These values are obtained in different ground conditions, different hydraulic drilling machines and drill bits by digitalization method^19,29,30^. The digitalization results illustrate that the variations of drilling speed measured by factual drilling data are compatible and consistent with the properties of different geomaterials from different ground conditions, different hydraulic drilling machines and drill bits.

### Statistical digitalization results for estimating the strength property of drilled geomaterials

According to the British and European Standard^31^, the drilling speed is related to the mechanical strength of drilled formation. Elkatatny proposed that the drilling speed plays a key role during the drilling process^32^. In this paper, the above results show that the constant drilling speed can represent the strength property of drilled geomaterials. The paralleled site loggings verify the accuracy of constant drilling speed from factual field data. Further discussions on the comparison between the constant drilling speeds and the traditional test results of mechanical strength properties are presented in the following.

**Figure 9:**
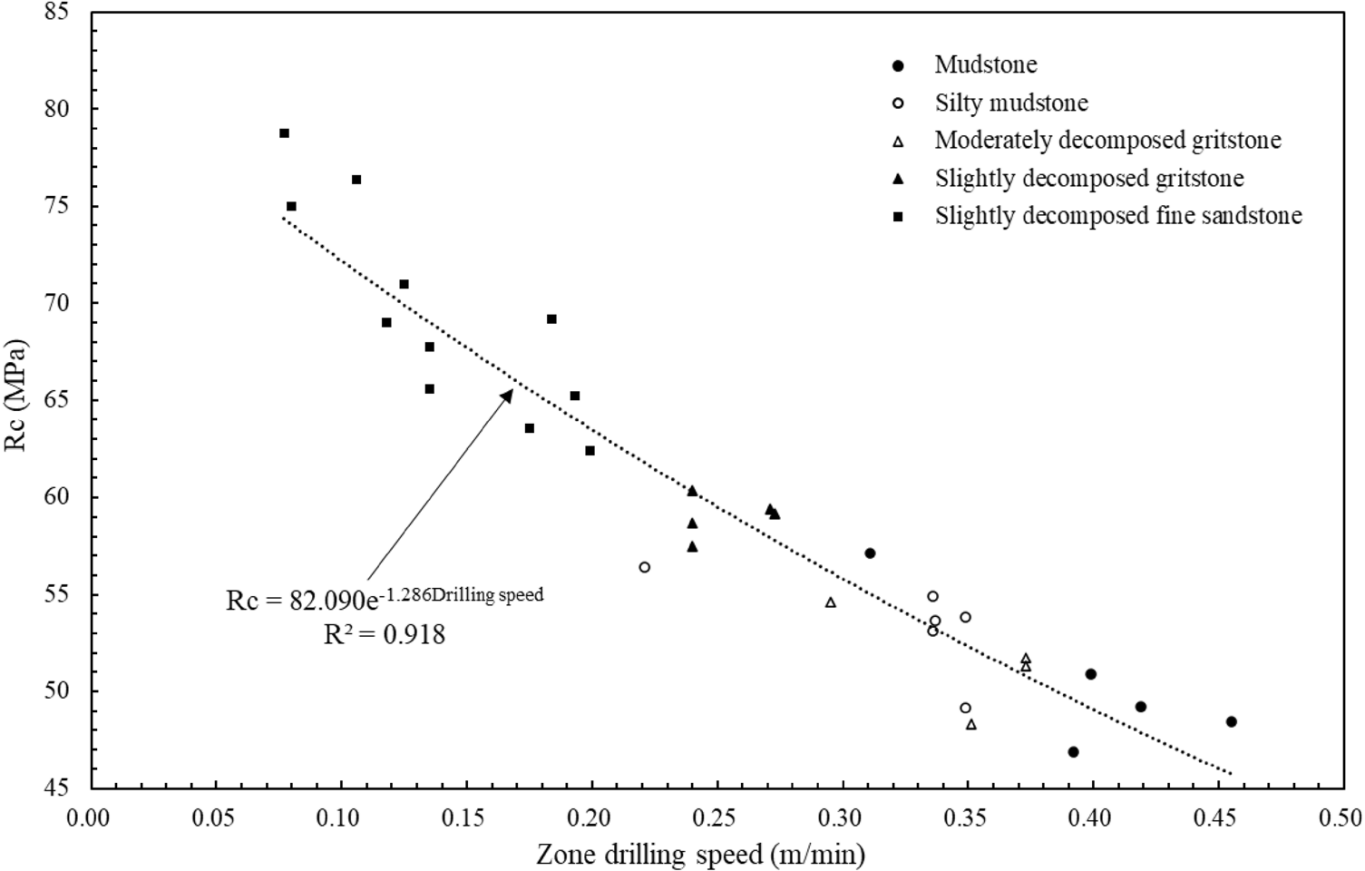
Comparison between the rock uniaxial compressive strength ${R}_{c}$ from laboratory test and the corresponding zone drilling speed from digitalization results.

The uniaxial compressive strength is the classical strength parameter for measuring the rock mass basic property. The uniaxial compressive strength of the core samples along this drillhole was obtained by laboratory test. Thirty-one core samples from depths 11.9 to 108.0 m were tested with GB/T 50218-2014 standard method. The beginning depth and ending depth of each core sample with a height of 10 cm were recorded on site. The depth of each specimen corresponds to one or several linear zones with different thicknesses at the same depth. The weighted drilling speeds of one or several linear zones at the same depth are compared with the uniaxial compressive strength test results, as shown in Fig. 9. The core samples of slightly decomposed fine sandstone with the lowest drilling speed have the highest values of uniaxial compressive strength. Conversely, the lowest values of uniaxial compressive strength are core samples of mudstone with the highest drilling speed among the rock strata. The results indicate that the values of uniaxial compressive strength decrease as the corresponding drilling speed increases. The regression equation in Fig. 9 can be expressed as Eq. (1). With this equation, the estimated values of uniaxial compressive strength by digitalization results can be obtained by the corresponding drilling speed.

1 $${\text{Uniaxial compressive strength (MPa) }} = { 82}{{.090}} \times e^{{ - 1.{286} \times {\text{Drilling speed (m/min)}}}}$$

Currently, effective methods to obtain the rock strength property in real-time on-site are scarce^33^. According to Eq. (1), the constant drilling speeds can estimate the strength property of the drilled geomaterials. The estimated values are consistent with the uniaxial compressive strength by traditional laboratory tests. Therefore, the constant drilling speeds provide the prediction of strength property by in-situ factual field data. The original factual data is practical and efficient to record in real-time series on site. The prediction result is accurate and applicable for siliciclastic sedimentary rocks along this 108 m deep drillhole. To build a generalized prediction method based on factual drilling data for wider applications, more studies on various geomaterial types, drilling machines, and drilling conditions are required.

### Statistical digitalization results for profiling the geomaterial strength quality designation

**Figure 10:**
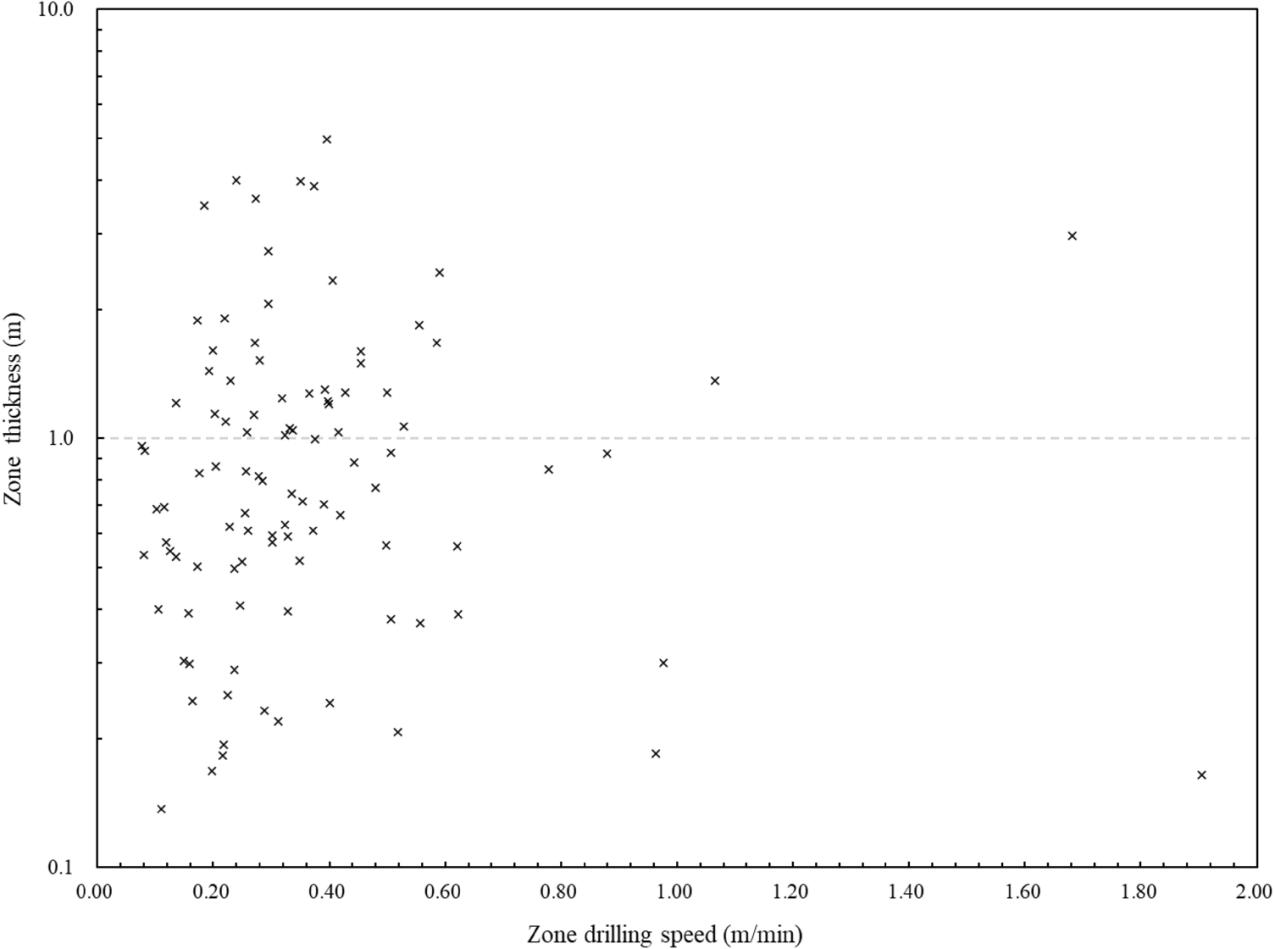
Variations of zone thickness with their corresponding zone drilling speed.

The digitalization data can also show the zoning results of the geomaterial strength quality designation by the similar method with rock quality designation (RQD). In classical rock mechanics, RQD can only represent the degree of fracturing in a rock core and cannot represent the strength and grade of weathering of the rock blocks because of its geometrical definition as follows. RQD is the borehole core recovery percentage incorporating pieces of solid core that are longer than 100 mm in length measured along the centerline of the core^34^. Each solid core piece is defined as piece greater than 100 mm between natural fractures. The solid core pieces can have high variations in mechanical strength (such as UCS). Therefore, a new parameter of constant drilling speed and zone thickness is needed to show the geomaterial strength profiles. Figure 10 illustrates the thickness of the 107 linear zones with the corresponding drilling speeds.

For the soils to sedimentary rocks along the drillhole, the zone thicknesses range from 0.137 to 4.970 m, andthe average and median values are 1.010 m and 0.746 m, respectively. The thickness of each zone and its drilling speed can be arranged to form the n-pair array from smallest to largest: $(V_{DPM1} ,H_{1} )$,$(V_{DPM2} ,H_{2} )$,…, $(V_{DPMn} ,H_{n} )$ where $V{}_{DPM1} \le V{}_{DPM2} \le \cdots \le V{}_{DPMn}$. Based on the digitalization data along the drillhole, the $RQD(V_{DPM} )$ can be defined as the percentage ratio of accumulated zone thicknesses with the drilling speed less than a given value over the whole drilling depth:

2 $$RQD(V_{DPM} ) = \frac{{\sum\nolimits_{i = 1}^{K} {H_{i} } }}{{\sum\nolimits_{i = 1}^{n} {H_{i} } }}$$

where $$K = 1,2,3, \dots,n$$; and $$\sum\nolimits_{i = 1}^{n} {H_{i} } = {108}{{.062 m}}$$.

**Figure 11:**
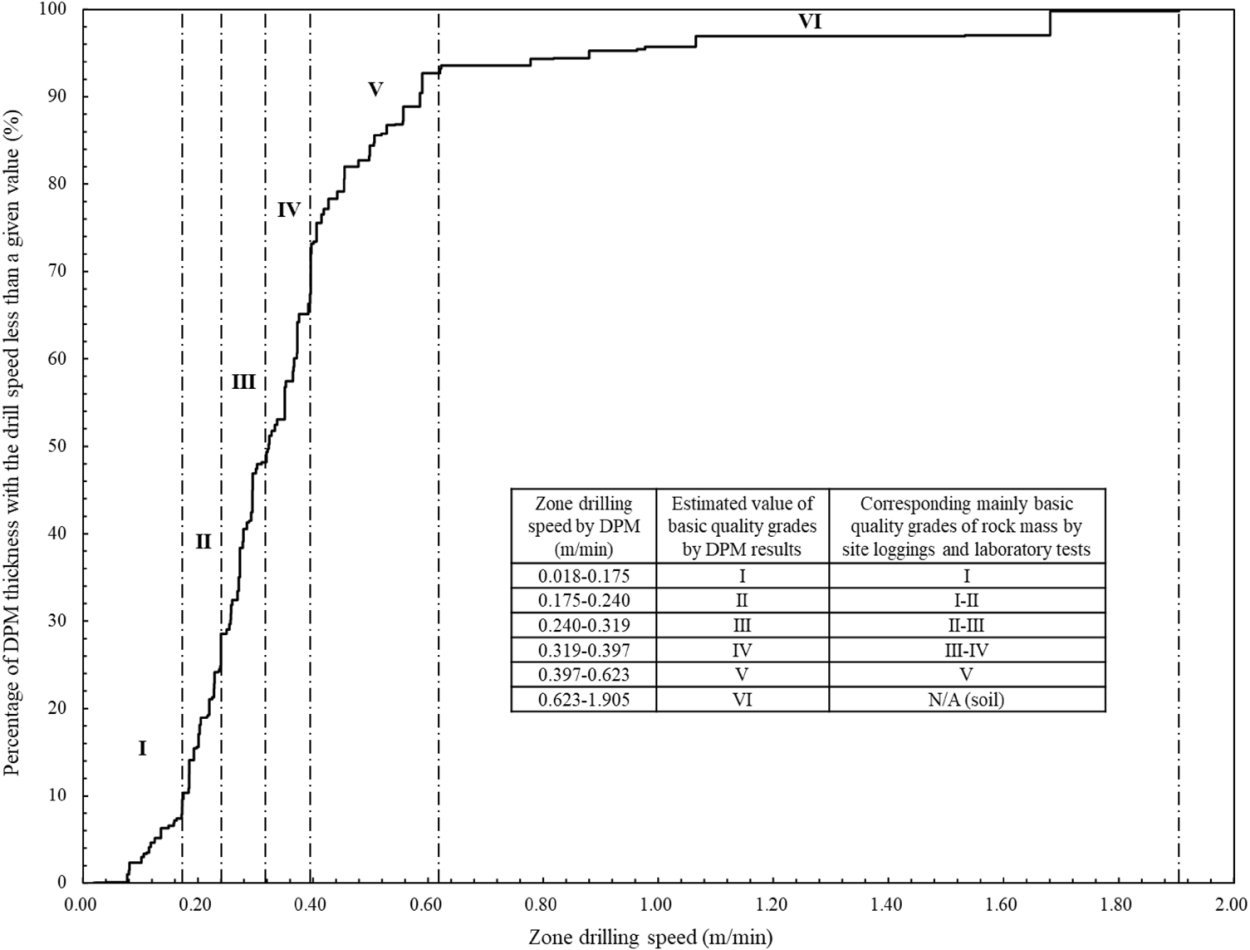
Distributions of the digitalization zone thickness with drilling speed (${\mathrm{RQD}}_{\mathrm{DPM}}$) for statistically profiling the geomaterial strength.

**Figure 12:**
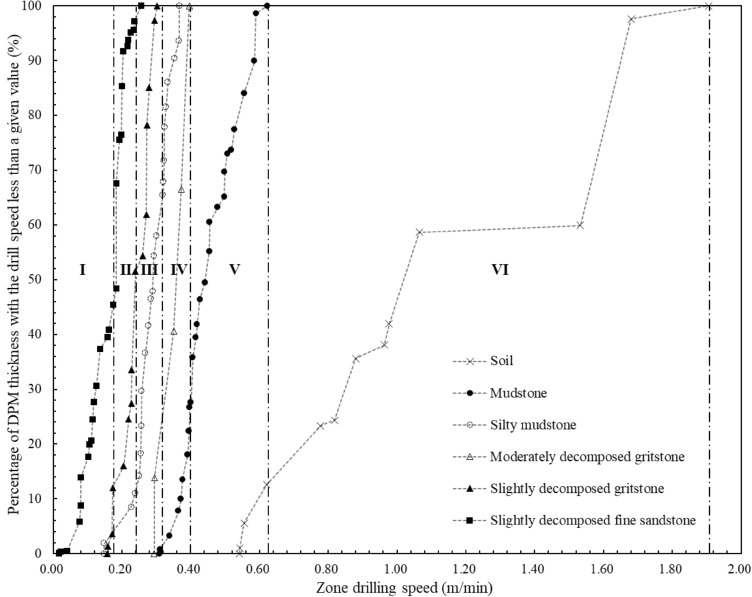
Distributions of the digitalization zone thickness with drilling speed (${\mathrm{RQD}}_{\mathrm{DPM}}$) for statistically profiling each of the 6 types of geomaterials.

**Figure 13:**
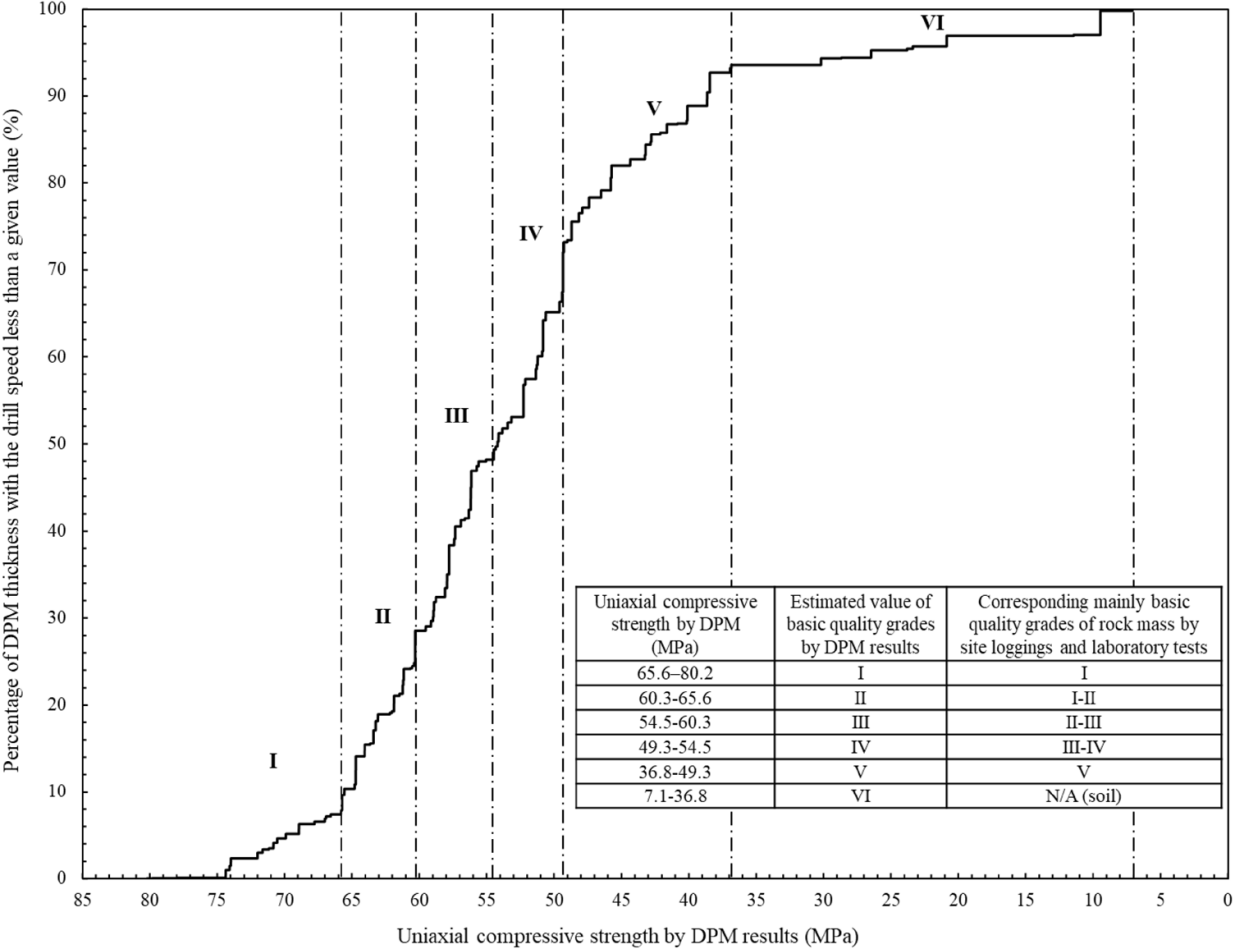
Distributions of the digitalization zone thickness with UCS for statistically profiling the geomaterial strength.

**Figure 14:**
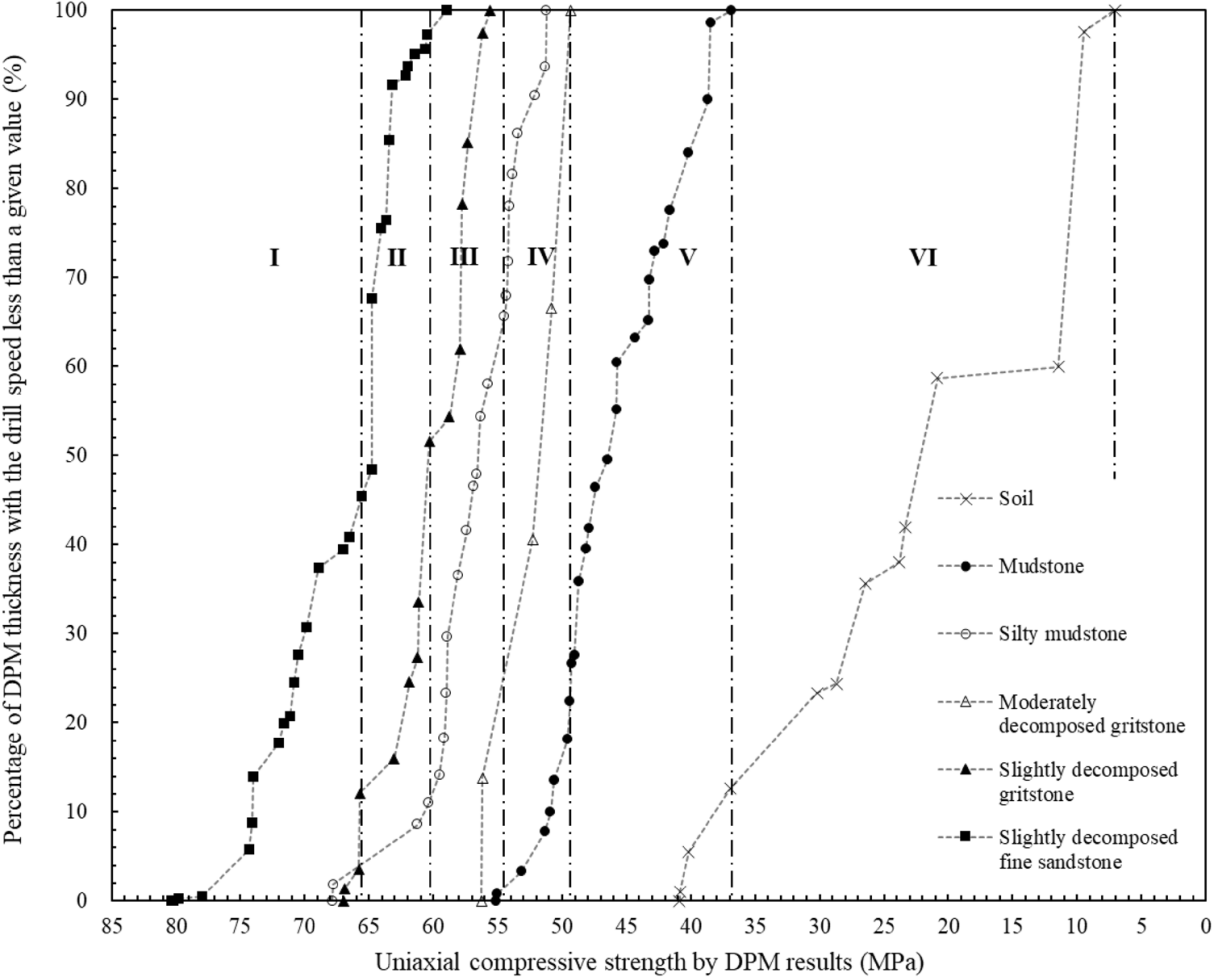
Distributions of the digitalization zone thickness with UCS for statistically profiling each of the 6 types of geomaterials.

$RQD(V_{DPM} )$ in this paper is not purely an extension of the traditional geometrical RQD method. It is a new parameter of constant drilling speed and zone thickness with the similar calculation method of RQD. The values of $RQD(V_{DPM} )$ and the associated zone drilling speeds can indicate the zoning results of the geomaterial strength profiles. It can be a complement to traditional in-situ geological investigation methods. Figure 11 shows the distributions of the digitalization zone thickness with drilling speed for statistically profiling the geomaterial strength. The vertical axis is the $RQD(V_{DPM} )$ value from Eq. (2) and the horizontal axis is the corresponding zone drilling speed. Figure 12 shows the detailed distributions for profiling each of the six types of geomaterials along the drillhole. The values of $RQD(V_{DPM} )$ and the associated uniaxial compressive strength by digitalization results are also presented to show the zoning results of the geomaterial strength profiles. Figure 13 shows the distributions of the digitalization zone thickness with uniaxial compressive strength by digitalization results for statistically profiling the geomaterial strength. The vertical axis is the $RQD(V_{DPM} )$ value from Eq. (2) and the horizontal axis is the corresponding uniaxial compressive strength by digitalization results from Eq. (1). Figure 14 shows the detailed distributions for profiling each of the six types of geomaterials along the drillhole.

From the similar variation patterns, the curves in Figs. 11 and 13 can be divided into the following six sections as the drilling speed increases along the horizontal axis:

- Section I means the estimated value of basic strength quality grades by digitalization results is the first grade with the drilling speed varying from 0.018 to 0.175 m/min. The estimated values of uniaxial compressive strength by digitalization data are from 65.6 to 80.2 MPa. The percentage number of digitalization thickness with the drilling speed less than the upper bound value of this section (i.e., 0.175 m/min) is about 10.38%. The geomaterial of this section is mainly slightly decomposed fine sandstone as shown in Figs. 12 and 14, corresponding to the basic quality grades of I by site loggings and laboratory tests.
- Section II means the estimated value of basic strength quality grades by digitalization results is the second grade with the values of drilling speed varying from 0.175 to 0.240 m/min. The estimated values of uniaxial compressive strength by digitalization data are from 60.3 to 65.6 MPa and the values of $RQD(V_{DPM} )$ are from 10.38 to 28.55%. The section contains mainly slightly decomposed fine sandstone and slightly decomposed gritstone as shown in Figs. 12 and 14, corresponding to the basic quality grades of I and II by site loggings and laboratory tests.
- Section III means the estimated value of basic strength quality grades by digitalization results is the third grade with the values of drilling speed varying from 0.240 to 0.319 m/min. The estimated values of uniaxial compressive strength by digitalization data are from 54.5 to 60.3 MPa and the values of $RQD(V_{DPM} )$ are from 28.55 to 49.33%. The section contains mainly slightly decomposed gritstone and silty mudstone as shown in Figs. 12 and 14, corresponding to the basic quality grades of IIand III by site loggings and laboratory tests.
- Section IV means the estimated value of basic strength quality grades by digitalization results is the fourth grade with the values of drilling speed varying from 0.319 to 0.397 m/min. The estimated values of uniaxial compressive strength by digitalization data are from 49.3 to 54.5 MPa and the values of $RQD(V_{DPM} )$ are from 49.33 to 73.19%. The geomaterials of this section are mainly silty mudstone and moderately decomposed gritstone as shown in Figs. 12 and 14, corresponding to the basic quality grades of III and IV by site loggings and laboratory tests.
- Section V means the estimated value of basic strength quality grades by digitalization results is the fifth grade with the values of drilling speed varying from 0.397 to 0.623 m/min. The estimated values of uniaxial compressive strength by digitalization data are from 36.8 to 49.3 MPa and the values of $RQD(V_{DPM} )$ are from 73.19 to 93.56%. The geomaterial of this section is mainly mudstone as shown in Figs. 12 and 14, corresponding to the basic quality grades of V by site loggings and laboratory tests.
- Section VI means the estimated value of basic strength quality grades by digitalization results is the sixth grade with the values of drilling speed varying from 0.623 to 1.905 m/min. The estimated values of uniaxial compressive strength by digitalization data are from 7.1 to 36.8 MPa and the values of $RQD(V_{DPM} )$ are from 93.56 to 100.00%. The geomaterial of this section is soil as shown in Figs. 12 and 14.

The criteria for basic strength quality classification of rock masses from the standard GB/T 50218-2014 and the digitalization method are further summarized in Table 2. In addition, the percentage distribution of zone thicknesses with the six basic quality grades can be measured with digitalization data for the different ground geomaterials. In Table 3, among the total thickness of 8.305 m for soil layers along the drillhole, 83.8% of soil corresponds to the basic quality grades of VI determined by digitalization data. 77.6% of mudstone along the drillhole belongs to the grade of V. 86.2% of moderately decomposed gritstone is the grade of IV. Most of the silty mudstone, which has a total thickness of 16.372 m, corresponds to the basic quality grades of III and IV. Most of the slightly decomposed gritstone corresponds to the basic quality grades of IIand III. For slightly decomposed fine sandstone, which has a total thickness of 18.128 m along the drillhole, 97.2% corresponds to the basic quality grades of I and II.

**Table 2:** Criteria for basic strength quality classification of rock masses or ground geomaterials from the standard GB/T 50218-2014 and digitalization method.

Criteria for basic quality classification of rock masses from GB/T 50218-2014				Criteria for basic quality classification of the ground geomaterials by DPM method for this site			
Basic quality grade	Uniaxial compressive strength (MPa)	Intactness index of rock mass	Basic quality index of rock mass (BQ)	Basic quality grade	Zone drilling speed (m/min)	Uniaxial compressive strength by DPM (MPa)	$RQD(V_{DPM} )$ (%)
I	> 60	> 0.75	> 550	I	0.018–0.175	65.6–80.2	0.00–10.38
II	> 60 60–30	0.75–0.55 > 0.75	550–451	II	0.175–0.240	60.3–65.6	10.38–28.55
III	> 60 60–30 30–15	0.55–0.35 0.75–0.55 > 0.75	450–351	III	0.240–0.319	54.5–60.3	28.55–49.33
IV	> 60 60–30 30–15 15–5	0.35–0.15 0.55–0.15 0.75–0.35 > 0.55	350–251	IV	0.319–0.397	49.3–54.5	49.33–73.19
V	30–15 15–5 ≤ 5	0.35–0.15 0.55–0.15 ≤ 0.15	≤ 250	V	0.397–0.623	36.8–49.3	73.19–93.56
Soil	N/A	N/A	N/A	VI	0.623–1.905	7.1–36.8	93.56–100.00

**Table 3:** Percentage distribution of zone thicknesses with the six basic strength quality grades for the ground geomaterials measured with digitalization data.

Type of geomaterial	Thickness distribution (%) of the six quality grades determined by DPM						
	I (%)	II(%)	III (%)	IV (%)	V (%)	VI (%)	Total thickness (m)
Soil	0.0	0.0	0.0	0.0	16.2	83.8	8.305
Mudstone	0.0	0.0	0.8	21.6	77.6	0.0	28.199
Silty mudstone	1.8	9.2	47.0	42.0	0.0	0.0	16.372
Moderately decomposed gritstone	0.0	0.0	13.8	86.2	0.0	0.0	14.895
Slightly decomposed gritstone	12.1	39.5	48.4	0.0	0.0	0.0	22.163
Slightly decomposed fine sandstone	45.4	51.8	2.8	0.0	0.0	0.0	18.128
Overall	10.4	18.2	19.6	23.9	21.5	6.4	108.062

**Figure 15:**
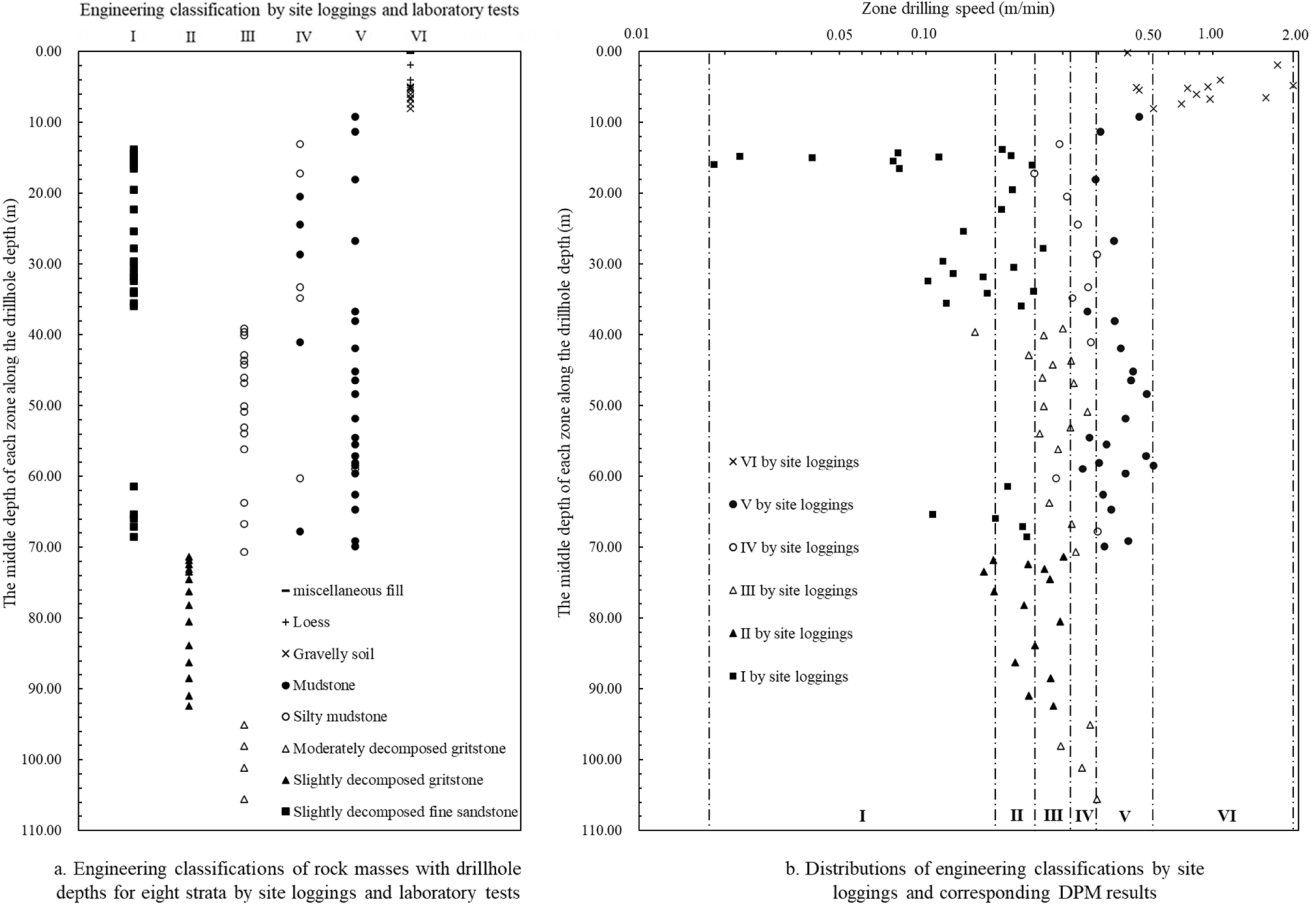
Comparison between engineering classification grades determined by digitalization data and manual loggings along the drillhole.

Figure 15 shows the comparison between engineering classification grades determined by digitalization data and manual loggings along the drillhole. The basic quality grades of rock masses determined by manual loggings with drillhole depths for different geomaterials are shown in Fig. 15a. The grades of I to V represent the laboratory test results for rock mass from the method of GB/T 50218-2014, and VI represents the soil layers. The distributions of the six sections for engineering classifications by digitalization data and the corresponding manual loggings are presented in Fig. 15b.

**Figure 16:**
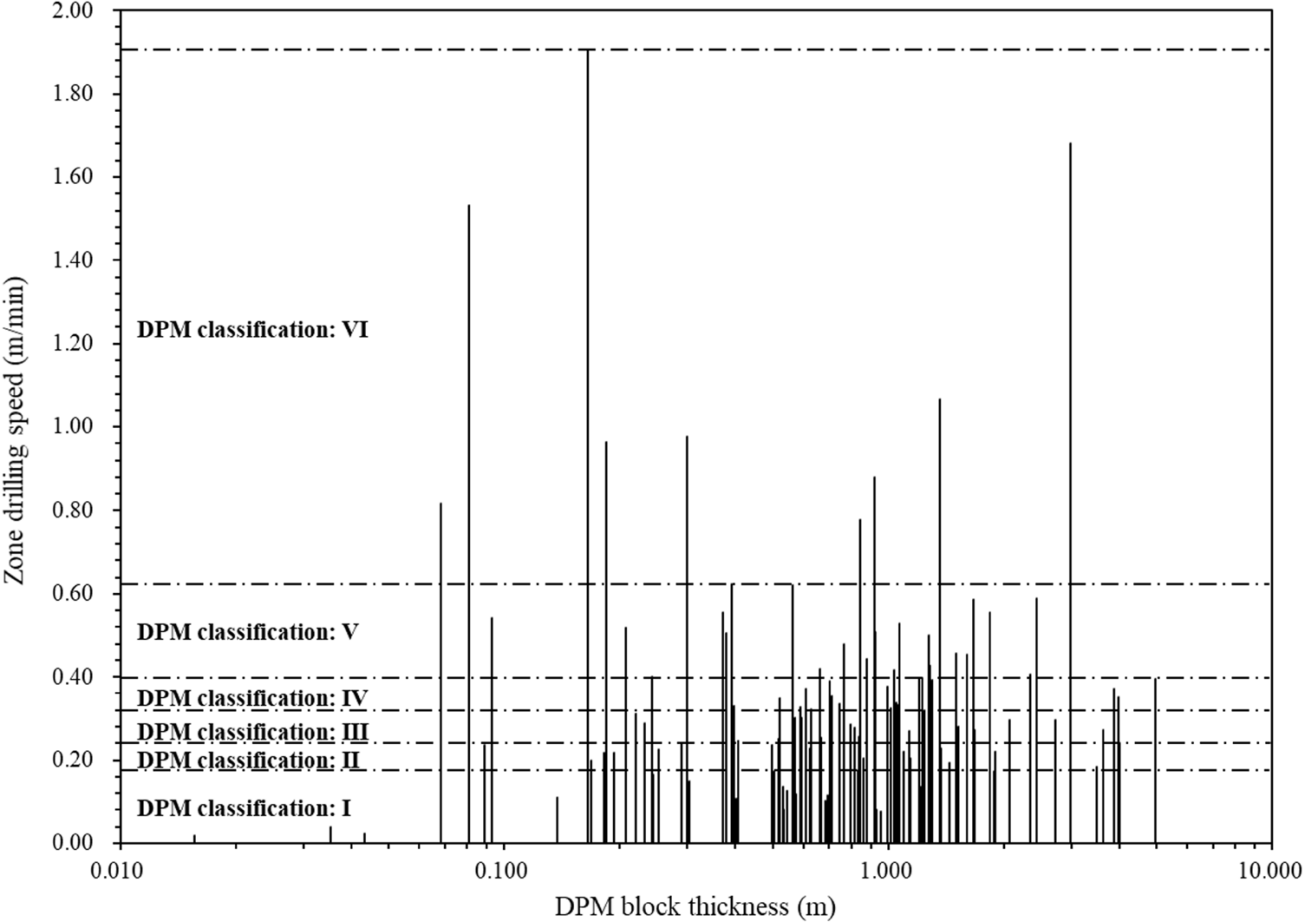
Distributions of drilling speed with its linear zone thickness.

For this drillhole, the strength quality classification grades of the drilled geomaterials are shown in Fig. 16. The digitalization results show that the constant drilling speeds are correlated with the uniaxial compressive strength, the rock quality designation, and the six basic strength quality grades for classifying the strength quality of superficial deposits to sedimentary rocks. The results can upgrade the current drilling practice for in-situ testing of continuous and mechanical profiles.

## Conclusions

The paper proposes a new digitalization method for the utilization of drilling information. The digital factual data in real-time series along the 108 m deep drillhole is recorded and analyzed for continuously and cost-effectively obtaining the in-situ strength profiling of drilled geomaterials.

Total 107 linear zones are identified by constant drilling speeds. The lower constant drilling speed of one linear zone indicates a stronger homogeneous geomaterial zone or strata. The mudstone, moderately decomposed gritstone, silty mudstone, slightly decomposed gritstone, and the fine sandstone have the average constant drilling speeds of 0.452 m/min, 0.354 m/min, 0.290 m/min, 0.236 m/min, and 0.149 m/min, respectively. The linear zones with constant drilling speeds can accurately and effectively show the interface boundaries and spatial distributions of the 51 strata for 8 types of soils and sedimentary rocks. Each manual stratum by traditional site logging is represented with one or more constant mechanical zones. The digitalization results provide a more detailed profile of the geomaterial strength along the drillhole.

Furthermore, the uniaxial compressive strength of the rock cores from laboratory tests has the correlation equation $R_{c} \, = { 82}{{.090}} \times e^{{ - 1.{286} \times {\text{Drilling speed (m/min)}}}}$ with the drilling speed. Six strength quality grades are determined by the drilling speed, the uniaxial compressive strength $R_{c}$, and the rock quality designation. The drilling speed,$R_{c}$, and $RQD(V_{DPM} )$ have the following ranges: (1) for grade I, 0.018–0.175 m/min, 65.6–80.2 MPa, and 0.00–10.38%; (2) for grade II, 0.175–0.240 m/min, 60.3–65.6 MPa, and 10.38–28.55%; (3) for grade III, 0.240–0.319 m/min, 54.5–60.3 MPa, and 28.55–49.33%; (4) for grade IV, 0.319–0.397 m/min, 49.3–54.5 MPa, and 49.33–73.19%; (5) for grade V, 0.397–0.623 m/min, 36.8–49.3 MPa, and 73.19–93.56%; (6) for grade VI, 0.623–1.905 m/min, 7.1–36.8 MPa, and 93.56–100.00%, respectively. The thickness distributions of each strength quality grades by digitalization data can also be obtained as 10.4%, 18.2%, 19.6%, 23.9%, 21.5%, and 6.4% for the quality grades I to VI, respectively.

The analysis results show that digitalization data can offer an additional, continuous and quantitative reference for the detailed spatial distributions of superficial deposits to siliciclastic sedimentary rocks and the associated in-situ mechanical profiling. This study offers a cost-effective method to upgrade the current drilling practice for in-situ testing of geomaterial strength profile and spatial distribution. In addition, the digitalized drilling data can also be affected by different drill machines and drill bits. For the same drilled geomaterial, the constant drilling speed of a high-power drill machine or a new sharper drill bit should be greater than the constant drilling speed of a low-power drill machine or an old dull drill bit. For wider applications of the new method in future, more studies of standardized drilling machines and bits are needed to develop a standard in-situ ground investigation test. Furthermore, the result of the DPM strength profile along drillhole can be used to determine and predict the deformation and collapse of the geomaterials surround the drillhole.

It is believed that the paper will fill the gap of the utilization for massive drilling data in geophysics and geology, and it will also provide a continuous and cost-effective tool to record and analyze the factual data of current drilling projects.

## Data Availability

The data used in this study are publicly available at Figshare from https://doi.org/10.6084/m9.figshare.14979582.
